# Chronic Sleep Deprivation Causes Anxiety, Depression and Impaired Gut Barrier in Female Mice—Correlation Analysis from Fecal Microbiome and Metabolome

**DOI:** 10.3390/biomedicines12122654

**Published:** 2024-11-21

**Authors:** Lingyue Li, Zilin Meng, Yuebing Huang, Luyao Xu, Qianling Chen, Dongfang Qiao, Xia Yue

**Affiliations:** Guangzhou Key Laboratory of Forensic Multi-Omics for Precision Identification, School of Forensic Medicine, Southern Medical University, Guangzhou 510515, China; 279134162lilingyue2021@163.com (L.L.); 15815621217@163.com (Z.M.); yuebhuang@163.com (Y.H.); 13534430942@163.com (L.X.); singlingchan@163.com (Q.C.)

**Keywords:** sleep deprivation, anxiety-like behavior, depression-like behavior, gut microbiota, female, colon barrier

## Abstract

Background: Chronic sleep deprivation (CSD) plays an important role in mood disorders. However, the changes in the gut microbiota and metabolites associated with CSD-induced anxiety/depression-like behavior in female mice have not been determined. Due to the influence of endogenous hormone levels, females are more susceptible than males to negative emotions caused by sleep deprivation. Here, we aim to investigate how CSD changes the gut microbiota and behavior and uncover the relationship between CSD and gut microbiota and its metabolites in female mice. Methods: We used a 48-day sleep deprivation (SD) model using the modified multiple platform method (MMPM) to induce anxiety/depression-like behavior in female C57BL/6J mice and verified our results using the open field test, elevated plus maze, novel object recognition test, forced swim test, and tail suspension test. We collected fecal samples of mice for 16S rDNA sequencing and untargeted metabolomic analysis and colons for histopathological observation. We used Spearmen analysis to find the correlations between differential bacterial taxa, fecal metabolites, and behaviors. Results: Our study demonstrates that CSD induced anxiety/depressive-like behaviors in female mice. The results of 16S rDNA sequencing suggested that the relative abundance of the harmful bacteria *g_ Rothia*, *g_ Streptococcus*, *g_ Pantoea*, and *g_ Klebsiella* were significantly increased, while the beneficial bacteria *g_ Rikenella*, *g_ Eubacterium]-xylanophilum-group*, and *g_ Eisenbergiella* were significantly decreased after SD. Glycerophospholipid metabolism and glutathione metabolism were identified as key pathways in the fecal metabolism related to oxidative stress and inflammatory states of the intestine. Histological observation showed hyperplasia of epithelial cells, a decrease in goblet cells, and glandular atrophy of the colon in SD mice. There were correlations between some of the differential bacterial taxa, fecal metabolites, and behaviors. Conclusion: In summary, we found that CSD induced anxiety/depression-like behavior, caused gut microbiota dysbiosis, altered fecal metabolism, and damaged the colon barrier in female mice.

## 1. Introduction

The process of sleep is a physiological necessity for the brain to restore crucial functions such as emotion, cognition, and memory [1]. Chronic sleep deprivation (CSD) can lead to many psychiatric symptoms, and anxiety and depression are two of them [2]. A Global Sleep Satisfaction Trends Survey released in 2020 revealed that less than half (49%) of the 13,004 survey subjects from 13 countries were satisfied with their sleep quality [3]. There is evidence to suggest that the gut microbiota exerts important functions in the regulation of sleep [4]. Accordingly, sleep deprivation (SD) can induce gut microbiota dysbiosis in the human intestine and may further damage the intestinal barrier or disrupt the homeostasis of the human body [5,6].

The microbiota–gut–brain axis (MGB) plays a role in depression, as indicated by changes in the gut microbiota in a rodent model of stress-induced depression. Similarly, many clinical studies have demonstrated abnormalities in the composition of the gut microbiota in the feces of patients with depression, further suggesting the role of the microbiota–gut–brain axis in depression [7,8]. As time goes by, SD may not only damage the intestinal environment but also lead to a vicious cycle of insomnia, which brings a heavy burden to society. However, only a handful of studies have examined the interaction between the gut microbiota and SD-induced anxiety/depression-like behaviors in mice; therefore, more research is needed to explore the effects of CSD on the gut microbiota as well as on depression.

Due to the influence of endogenous hormone levels, women are almost twice as likely as men to have sleep problems and suffer from depression [9]. In addition, there is evidence that females are more susceptible than males to negative emotions caused by SD [10,11,12,13,14]. Current studies on SD have mostly used male animals as research subjects, while females have been excluded from many studies [15], so here, we used female mice to investigate SD-induced alterations in gut microbiota and anxiety/depression-like behaviors.

Therefore, in the present study, we used the modified multiple platform method (MMPM) to establish a CSD model and evaluated anxiety/depression-like behaviors in mice [1,16,17]. We co-applied bacterial 16S rDNA sequencing and fecal untargeted metabolomics to study the changes in the gut microbiota and the effects of the metabolites in CSD mice. Our findings provide new insights into the mechanisms by which SD leads to anxiety/depressive-like behaviors and serve as a wake-up call for human health problems related to SD and its triggering of anxiety/depressive-like behaviors.

## 2. Materials and Methods

### 2.1. Animals

Six-week-old specific-pathogen-free (SPF) female C57BL/6J mice weighing approximately 20–25 g were purchased from the Guangdong Medical Laboratory Animal Center (Foshan, China). The mice were housed in a temperature-controlled room (5/6 per cage) with a 12:12-h light/dark cycle (lights on at 08:00) and had access to food and water ad libitum. Before the experiments, the mice were allowed 2 weeks of acclimatization. During the last week of acclimation, the mice were gently handled by the experimenter for 3–5 min each day. All procedures were performed according to the National Institutes of Health Guide for the Care and Use of Laboratory Animals and the Ethics Committee of Animal Experiments of Guangzhou TopBiotech Co., Ltd. (Guangzhou, China). This study was approved by the Ethics Committee of Animal Experiments of Guangzhou TopBiotech Co., Ltd. (Approval code: TOP-IACUC-023-0116).

### 2.2. Sleep Deprivation Procedure

**Figure 1:**
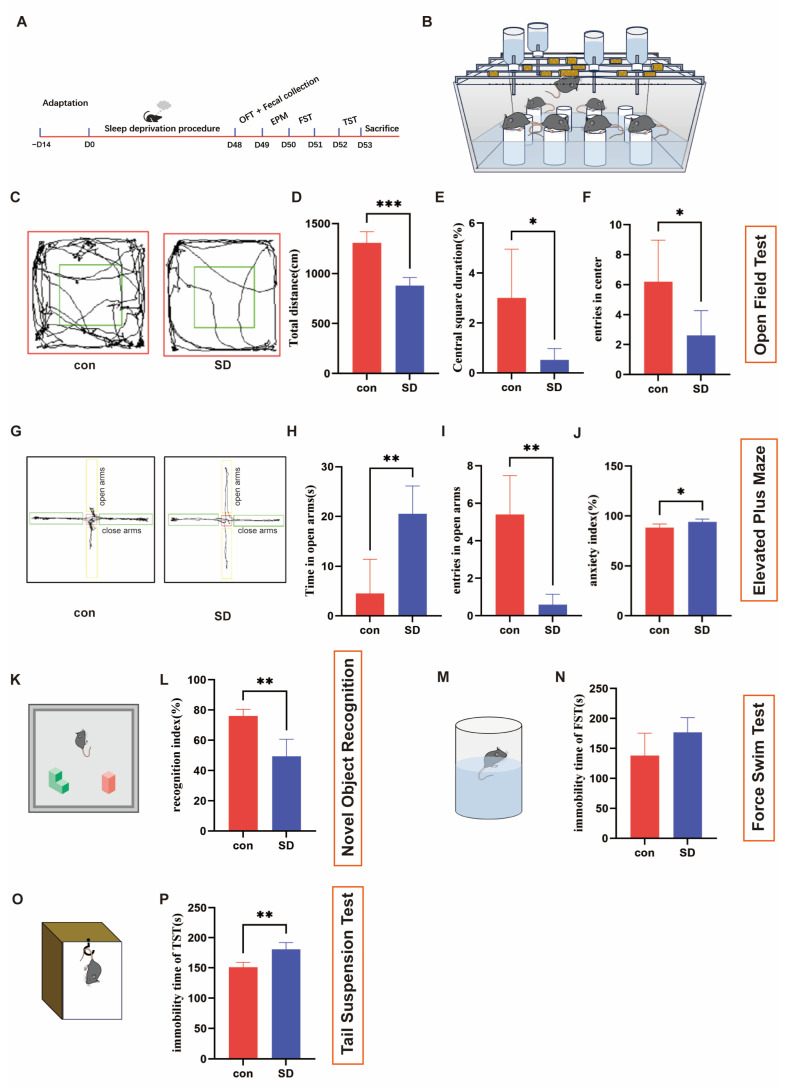
SD procedure and behavior test. (**A**) Schematic design of 48-d SD procedure and behavior test. (**B**) Diagram of the MMPM. (**C**) Representative tracking plot from the OFT. (**D**–**F**) Total distance (unpaired *t*-test, *n* = 4/5 per group), central square duration (unpaired *t*-test, *n* = 4 per group), and the number of entries in the center (unpaired *t*-test, *n* = 5 per group) during the OFT. (**G**) Representative track plot of the EPM test. (**H**–**J**) Time spent in the open arms (unpaired *t*-test), the number of entries in the open arms (unpaired *t*-test), and the anxiety index during the EPM test (unpaired *t*-test, *n* = 5 per group). (**K**) Diagram of the NORT. The green polyhedron represents familiar object, the red cube represents the novel object. (**L**) Recognition index of NORT (unpaired *t*-test, *n* = 4 per group). (**M**) Diagram of the FST. (**N**) Immobility time during FST (unpaired *t*-test, *n* = 5 per group). (**O**) Diagram of the TST. (**P**) Immobility time during TST (unpaired *t*-test, *n* = 5 per group). All data are presented as mean ± SEM. * *p* < 0.05, ** *p* < 0.01 and *** *p* < 0.001.

Mice were randomly divided into the SD group (*n* = 5) and the control group (*n* = 6). The MMPM was used to deprive animals of REM sleep. Briefly, 12 cylindrical platforms (d = 3 cm, h = 3 cm) were fixed in a polypropylene box (52 cm × 33 cm × 15 cm), and then the box was subsequently filled with water up to 1cm below the platforms. Food and water were available on the metal net in the box (Figure 1B). The SD group mice were placed in the box for acclimatization (1 h/day, 10:00–11:00) during the last three days of the habituation period. After 20 h of SD, the mice were taken out of the platform from 17:00 to 21:00 each day and placed in their home cages to allow them to recover for 4 h. SD lasted for 48 days. The mice were observed daily for clinical characteristics such as fatigue, lethargy, fear, irritability, and other behavioral changes. The mice of the control group were placed in their home cages.

### 2.3. Behavior Tests

After 48 days of SD, we quantified behavioral changes in the mice by using the open field test (OFT) [16], elevated plus maze (EPM) test [18], novel object recognition test (NORT) [18], force swim test (FST), and tail suspension test (TST) [19]. The animals were placed in the test room for at least 1 h before the behavioral tests to acclimate to the test environment. All the apparatuses were cleaned with 75% ethanol during behavioral testing to eliminate odors left by the previous mouse.

#### 2.3.1. Open Field Test (OFT)

The OFT was performed in a box (40 cm × 40 cm × 40 cm). At the beginning of the test, each mouse was placed in a corner and data were recorded for 5 min using a camera above the box. The box was cleaned with 75% alcohol after each trial. The total distance traveled, percentage of time spent in the central area (the central area of the open field apparatus was 50% of the total area), and the number of times the mouse entered the central area were analyzed via Smart 3.0 software to assess anxiety/depression-like behavior.

#### 2.3.2. Elevated Plus Maze (EPM)

The EPM test was performed in the EPM apparatus, which consisted of two opposing open arms (30 cm × 6 cm) and two opposing closed arms (30 cm × 6 cm, with 15 cm high walls) that extended from a central platform (6 cm × 6 cm) at 90°, and an elevated platform 50 cm from the floor. Initially, the mouse was placed on the central platform and faced the open arm. The maze was cleaned with 75% alcohol after each trial. The time spent in the open arms and closed arms, as well as the number of entries into the open arms, were recorded by a camera and analyzed by Smart 3.0 software to assess anxiety/depression-like behavior. The anxiety index was calculated according to [1]’s formula.

#### 2.3.3. Novel Object Recognition Test (NORT)

The NORT was performed in the same box as the OFT, including three phases (habituation, familiarization, and testing). Briefly, in the habitual phase, every mouse was allowed to explore the box (without any objects) for 10 min. During the familiar phase (1 h after habituation), the mice were allowed to explore two identical objects (plastic toy bricks) for 5 min. During the testing phase (1 h after familiarization), one of the familiar objects was replaced by a novel object (of a different color and shape), and the mice were allowed to explore for 5 min. The box and objects were cleaned with 75% alcohol after each exploration. The last two phases were recorded by a camera. The definition of exploration was the total time that a mouse touched the object with its mouth or nose within 2 cm of the object in close proximity. The time spent exploring these two objects during the testing phase was manually counted, and the recognition indices were calculated as follows: (novel object/(novel object + familiar object) × 100%).

#### 2.3.4. Forced Swim Test (FST)

The FST was performed in a transparent plexiglass cylinder (43 cm height × 20 cm diameter) filled with water to a height of 19 cm at a temperature of 25 °C. The test was continued for 6 min. The mouse was considered to be “immobile” when it became static in the water and only made small movements to keep its head on the surface of the water. The test lasted for 6 min, with the first 2 min being the pre-testing and the last 4 min being the testing phase. The immobility time in the test phase was manually counted by a person who was blinded to the groups. The FST is considered a valid method for assessing depressive-like behavior.

#### 2.3.5. Tail Suspension Test

The TST was performed in a dark hollow plastic case (20 cm × 20 cm × 30 cm) with a hook on the top. Mice were individually suspended on the hook by their tails with tape (1 cm distance from the tail tip) for 6 min. The mouse was considered to be “immobile” when it became static or only made small movements passively. The test lasted for 6 min, with the first 2 min being the pre-testing and the last 4 min being the testing phase. The immobility time in the test phase was manually counted by a person who was blinded to the groups. The tail suspension test was also used to assess depressive-like behavior.

### 2.4. Histopathological Observation

To observe the histological structure, colon tissues were embedded in paraffin after fixation with 4% paraformaldehyde. The tissues were subsequently sectioned into ultrathin 3 μm pieces and stained with hematoxylin and eosin (HE). To observe goblet cells, colon tissue sections were stained with an Alcian blue periodic acid Schiff stain kit (G1285, Solarbio, Beijing, China) according to the manufacturer’s instructions. The number of goblet cells was manually counted, and the area of interest (AOI) was measured using image-pro plus v.6.0.

### 2.5. Fecal Sample Collection

Fecal samples were collected after the OFT. The fecal pellets were collected from the box, placed into individual 1.5 mL sterile and enzyme-free microfuge tubes, and frozen at −80 °C until microbiome and metabolome analysis.

### 2.6. Fecal 16S rDNA Sequencing

The E.Z.N.A.^®^ stool DNA Kit (D4015, Omega, Inc., Norcross, GA, USA) was used to extract total microbial DNA from fecal samples (*n* = 6/5, control/SD), following the manufacturer’s instructions. The V3-V4 region of the 16S rRNA gene was amplified using primers 341F and 805R. Subsequently, PCR products were purified with AMPure XT beads (Beckman Coulter Genomics, Danvers, MA, USA) and quantified using Qubit (Invitrogen, Carlsbad, CA, USA). Amplicon pools were prepared for sequencing and assessed for size and quantity on an Agilent 2100 Bioanalyzer (Agilent, Santa Clara, CA, USA) and with the Library Quantification Kit for Illumina (Kapa Biosciences, Woburn, MA, USA), respectively. Finally, libraries were sequenced on the NovaSeq PE250 platform.

The samples were sequenced on an Illumina NovaSeq platform following the manufacturer’s recommendations and provided by LC-Bio. Quality filtering of the raw reads was performed using specific filtering conditions to obtain high-quality clean tags according to fqtrim (v0.94). Chimeric sequences were filtered using Vsearch software (v2.3.4). Alpha diversity and beta diversity were analyzed using the QIIME2 process, and visualizations were generated with R (v3.5.2). The species annotations in the sequence alignment were performed using BLAST, with SILVA and NT-16S as the alignment databases.

### 2.7. Untargeted Metabolomic Analysis

The protocol was conducted following previously established methods with minor adjustments [20]. Initially, fecal samples (*n* = 6/5, control/SD) were individually pulverized using liquid nitrogen, and the resulting homogenate was resuspended in prechilled 80% methanol (LC-MS Grade, Thermo Fisher, Waltham, MA, USA) by thorough vortexing. Subsequently, the samples were incubated on ice for 5 min, followed by centrifugation at 15,000× *g* and 4 °C for a duration of 20 min. A portion of the supernatant was then diluted to a final concentration of 53% methanol using LC-MS grade water (Merck, Darmstadt, Germany). The resultant mixture was subsequently transferred to a fresh Eppendorf tube and subjected to further centrifugation at 15,000× *g* and 4 °C for an additional period of 20 min. Finally, the supernatant obtained after centrifugation was injected into the LC-MS/MS system (Vanquish UHPLC/Q Exactive™ HF-X).

The samples were injected onto a Hypesil Gold column (100 × 2.1 mm, 1.9 μm, Thermo Fisher, Waltham, MA, USA) using a linear gradient over a period of 12 min at a flow rate of 0.2 mL/min. For the positive polarity mode, eluent A (0.1% FA in water, LC-MS Grade, Thermo Fisher, Waltham, MA, USA) and eluent B (methanol) were used as the mobile phases. For the negative polarity mode, eluent A (5 mM ammonium acetate, LC-MS Grade, Thermo Fisher, Waltham, MA, USA; pH 9.0) and eluent B (methanol) were employed as the mobile phases. The solvent gradient was programmed as follows: initial conditions of 2% B for 1.5 min, followed by an increase from 2% to 85% B over a period of 3 min, then an increase from 85% to 100% B over a period of10 min, subsequently maintaining at 100% B for another 10 s before returning to initial conditions within 10 milliseconds and equilibrating for another 12 min with 2% B. The Q ExactiveTM HF-X mass spectrometer was operated in both positive and negative polarity modes with spray voltage set at 3.5 kV, capillary temperature maintained at 320 °C, sheath gas flow rate set at 35 psi, aux gas flow rate maintained at 10 L/min, S-lens RF level set to 60, and Aux gas heater temperature controlled at 350 °C.

The raw data files generated by UHPLC-MS/MS were processed using Compound Discoverer 3.1 (CD3.1, Thermo Fisher, Waltham, MA, USA) for peak alignment, peak picking, and quantitation of each metabolite. Subsequently, the main parameters, including retention time tolerance, actual mass tolerance, signal intensity tolerance, signal/noise ratio, and minimum intensity, were established. Following this step, the peak intensities were normalized to the total spectral intensity. The normalized data were utilized to predict the molecular formula based on additive ions, molecular ion peaks, and fragment ions. Furthermore, the matching of peaks with mzCloud (https://www.mzcloud.org/, accessed on 12 August 2022), mzVault, and MassList databases was performed to obtain accurate qualitative and relative quantitative results. Metabolites exhibiting VIP > 1 and *p*-value < 0.05, along with fold change ≥ 2 or FC ≤ 0.5 in principal components analysis (PCA) and partial least squares discriminant analysis (PLS-DA) were considered as differential metabolites. PCA and PLS-DA analyses were conducted using metaX software (Version 2.0.0), which is a comprehensive tool for processing metabolomics data; statistical significance was calculated through a *t*-test utilizing a threshold of *p*-value < 0.05. Volcano plots based on log2 (FoldChange) and −log10 (*p*-value) values of metabolites obtained from the ggplot2 package in R language facilitated filtering of metabolites of interest. The annotation process involved the utilization of the KEGG database (https://www.genome.jp/kegg/pathway.html, accessed on 12 August 2022), HMDB database (https://hmdb.ca/metabolites, accessed on 12 August 2022), and LIPIDMaps database(http://www.lipidmaps.org/, accessed on 12 August 2022) for identification.

### 2.8. Statistical Analysis

Statistical analysis was performed using GraphPad Prism version 9.0 (GraphPad Software, San Diego, CA, USA). All data are presented as the mean ± SEM and were analyzed by *t*-tests unless otherwise stated. A *p*-value < 0.05 was considered statistically significant, * *p* < 0.05, ** *p* < 0.01, *** *p* < 0.001, **** *p* < 0.0001. Spearman’s correlation analysis was used to determine the relationships among behaviors, gut microbiota, and fecal metabolites. *p* < 0.05 was considered significant.

## 3. Result

### 3.1. SD Induced Anxiety/Depressive-like Behavior in Mice

To assess behavioral changes in mice after 48 days of SD, we performed behavioral tests. In the OFT (Figure 1C–F), we found that SD mice moved less in total distance (t = 6.321, *p* = 0.0004) and spent less time in the central area (t = 2.465, *p* = 0.0488) and decreased entries into the central area (t = 2.484, *p* = 0.0379) than the control mice. In the EPM (Figure 1G–J) test, the times SD mice entered the open arms was significantly less than control mice (t = 5.004, *p* = 0.0010), but the time SD mice spent in the open arms was longer than the control mice (t = 4.045, *p* = 0.0037). The anxiety index of the SD mice was significantly higher compared to the control mice according to the formula (t = 2.756, *p* = 0.0248). In the NORT (Figure 1K,L), the recognition index of SD mice was significantly lower than the control mice (t = 4.339, *p* = 0.0046). In the FST (Figure 1M,N), the immobility time of SD mice was higher than control mice but with no statistical differences between the two groups (t = 1.982, *p* = 0.0899). In the TST (Figure 1O,P), the immobility time of SD mice was significantly higher than control mice (t = 4.751, *p* = 0.0014). These results suggest that 48 d of sleep deprivation induced anxiety/depressive-like behavior in mice.

### 3.2. SD Disrupts the Gut Barrier

**Figure 2:**
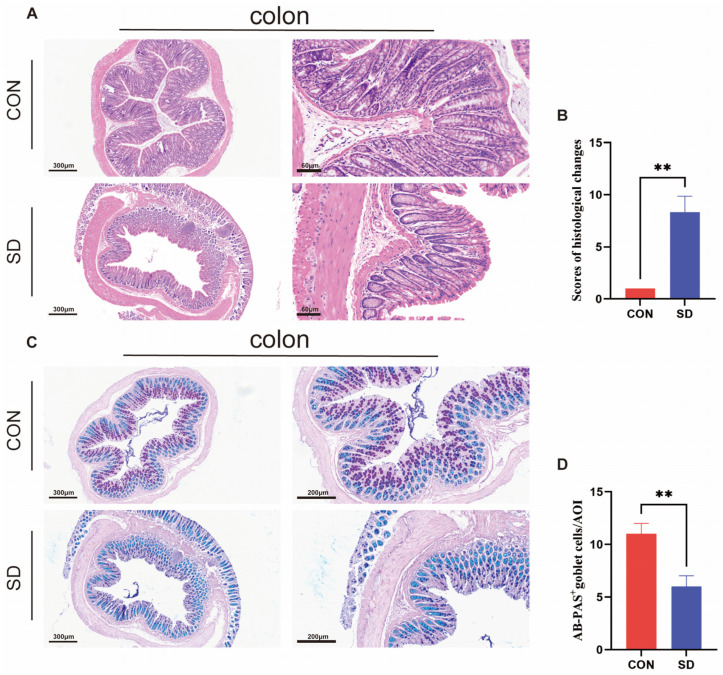
Colon pathological analysis. (**A**) Hematoxylin and eosin (H&E) staining. Bar = 300 μm and 60 μm. (**B**) Scores of histological changes in H&E staining (*n* = 3). (**C**) Alcian Blue Periodic Acid Schiff (AB-PAS) Staining. Bar = 300 μm and 200 μm. (**D**) Goblet cell counting of AB-PAS staining (*n* = 3). All data are presented as mean ± SEM. ** *p* < 0.01.

HE staining showed alterations in the colonic tissues in the SD group compared to the control group, mainly in the form of epithelial cell hyperplasia, goblet cell reduction, and glandular atrophy (Figure 2A,B). Histological changes were scored according to the following criteria: epithelial cell hyperplasia manifested by an increase in the number of epithelial cells per crypt or visible as crypt elongation (minimum: <25%, 1, mild: 25–35%, 2 or 3, moderate: 36–50%, 3 or 4, marked: >51%, 4 or 5), and goblet cell reduction (minimum: <20%, 1 or 2, mild: 21–35%, 2 or 3, moderate: 36–50%, 3 or 4, marked: >50%, 4). Glandular atrophy was characterized by a decrease in the number of crypts or a gland diameter smaller than the distance between the two glands and thickening of the base (minimum: 0, mild: 1, moderate: 2, marked: 3) [21]. AB-PAS staining showed a decreased number of goblet cells in the SD group (Figure 2C,D) (*p* < 0.05).

### 3.3. Sleep Deprivation Changed the Composition of the Gut Microbiota

**Figure 3:**
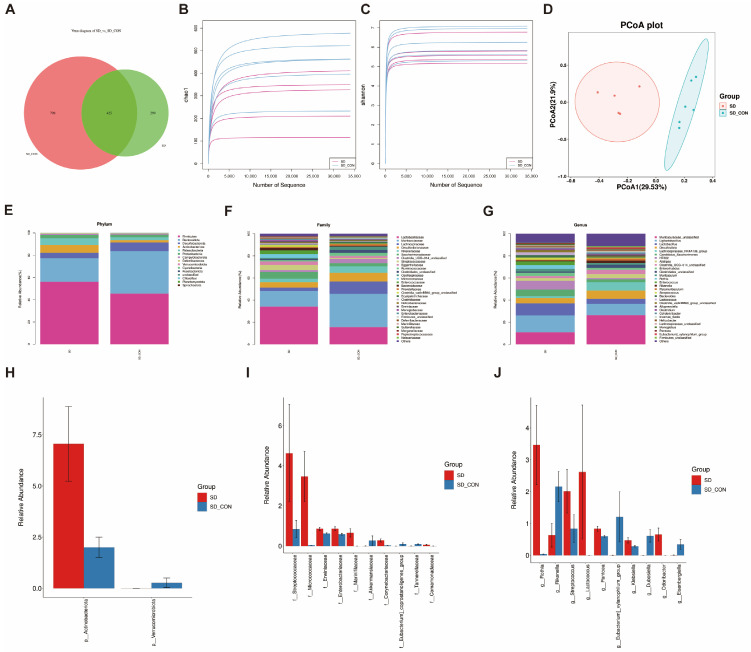
Fecal microbiome data analysis after SD. (**A**) Venn diagram. (**B**,**C**) In representative diagrams of alpha diversity, all alpha diversity indicators have no statistically significant differences. (**D**) Principal coordinates analysis (PcoA) plot using Bray–Curtis distance. (**E**) The ratio of relative abundances of phylum level. (**F**) The ratio of relative abundances of family level. (**G**) The ratio of relative abundances of genus level. (**H**) The top 10 species with a *p*-value less than 0.05 at the phylum level. (**I**) The top 10 species with a *p*-value less than 0.05 at the family level. (**J**) The top 10 species with a *p*-value less than 0.05 at the genus level.

**Figure 4:**
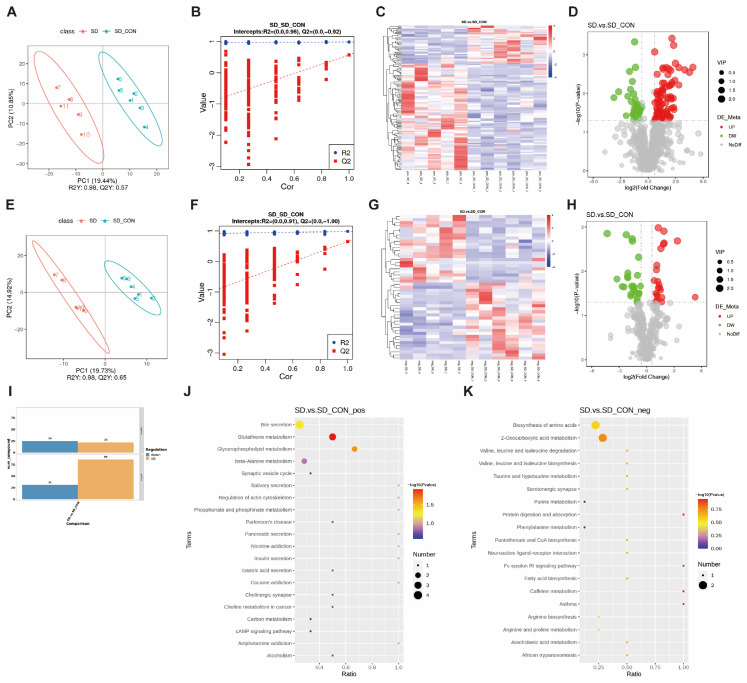
Fecal metabolomics after SD. (**A**) Score plot of PLS-DA model in positive ion model. (**B**) Permutation plot in positive ion model. (**C**) Heatmap graph of differential metabolites in positive ion model, the metabolites are clustered according to the similarity of the metabolite expression profiles. (**D**) Volcano plot in positive ion model, showing the distribution of differential metabolites. (**E**–**H**) Score plot of PLS-DA, permutation plot, heatmap graph, and volcano plot in negative ion model. (**I**) Differential metabolite statistics. (**J**,**K**) KEGG enrichment analysis of differential metabolites in positive ion model and negative ion model.

To explore how SD alters the gut microbiota composition, we employed 16S rDNA sequencing. A total of 576,110 raw reads were identified in 11 fecal samples (*n* = 6/5, control/SD). A Venn plot showed (Figure 3A) that there were 425 identical OTUs in the two groups, with another 798 OTUs exclusively in the SD-CON group and 299 OTUs exclusively in the SD group. Alpha diversity was mainly measured by the indices of Chao1, Observed species, Goods_coverage, Shannon, Simpson, and pielou-e to reflect species richness and homogeneity (Figure 3B,C), while none of the above indices were significant, indicating that SD did not significantly affect the diversity and richness of gut microbiota in mice. Next, β-diversity analysis was performed by principal coordinate analysis (PCoA) based on the Bray–Curtis distance at the OTU level. The result showed that SD altered the composition of gut microbiota in SD mice compared to controls (Figure 3D). The composition of gut microbiota was analyzed at the phylum, family, and genus levels (Figure 3E–G), and the gut microbiota of both the SD and control groups were mainly composed of *Firmicutes* and *Bacteroidota*. At the phylum level, the relative abundance of bacterial taxa, from high to low, was dominated by *Firmicutes*, *Bacteroidota*, *Desulfobacteroidota*, and *Actinobacteriota*. Among them, *Firmicutes* and *Bacteroidota* were predominant. Compared with the control group, the bacterial abundance of *Actinobacteriota* was increased, and the bacterial abundance of *Verrucomicrobiota* was decreased in the SD group (*p* < 0.05). The top 10 species with *p*-values less than 0.05 were selected to plot the histogram (Figure 4H–J). At the family level, the relative abundance of the families *Streptococcaceae*, *Micrococcaceae*, *Erwiniaceae*, and *Enterobacteriaceae* was significantly higher (*p* < 0.05) in the SD group compared with the control group, while the relative abundance of the family *Akkermansiaceae* was significantly lower. At the genus level, the relative abundance of the genera *Rothia*, *Streptococcus*, *Lactococcus*, *Pantoea*, and Klebsiella was significantly increased (*p* < 0.05) in the SD group, and the relative abundance of the genera *Rikenella*, *Eubacterium]_xylanophilum_group*, and *Eisenbergiella* was significantly decreased (*p* < 0.05).

### 3.4. SD Changed the Fecal Metabolome

There is increasing evidence that the gut microbiota plays an important role in generating active metabolites that may alter physiological functions. To explore these changes, we employed the LC-MS non-targeted metabolomic technique. PLS-DA analysis can reflect the differences between different groups to the greatest extent. The PLS-DA model showed a clear distinction between the SD group and the CON group, and the R2 was 0.98 in both positive and negative ion mode, which indicated that the model has a better explanatory rate (Figure 4A–E), but the Q2 was 0.57 and 0.65, respectively, which was usually better when the Q2 is closer to 1. Therefore, the prediction rate of this model was moderate. The model parameters R2 and Q2 were subjected to a permutation test, and the permutation test plot showed that Q2 was −0.92 and −1.00 in the positive and negative ion modes, indicating that the model was not overfitted and that the differential metabolite analysis was relatively accurate (Figure 4B,F). Based on the proximity of the metabolite expression profiles of the samples, the metabolites were clustered and analyzed to draw a heat map of differential metabolites, which could demonstrate the abundance differences of metabolites in the two groups. Red indicates the high-expressed metabolites, and blue indicates the low-expressed metabolites (Figure 4C,G). Using ratio ≥ 1.5 or ratio ≤ 0.677, *p*-value < 0.05, and VIP ≥ 1 as the screening conditions, a total of 164 differential metabolites were obtained, of which 108 were significantly up-regulated metabolites and 56 were significantly down-regulated metabolites (Figure 4D,H,I).

According to the classification of the HMDB database, it mainly included lipid metabolites, organic acids and their derivatives, organic nitrogen compounds, alkaloids and their derivatives, and benzene compounds, etc., and the most abundant of these metabolites were lipid metabolites. We identified six glycerophospholipid metabolites from the HMDB annotation classification and Lipidmaps annotation classification of metabolites (Table 1).

KEGG pathway enrichment analysis (Figure 4J,K) of the above differential metabolites revealed that the differential metabolite pathways between the SD and CON groups were mainly focused on glycerophospholipid metabolism, glutathione metabolism, bile secretion, β-alanine metabolism, amino acid synthesis, and 2-oxocarboxylic acid metabolism. Among them, glutathione metabolism and glycerophospholipid metabolism had the most significant effects, and thus, these two pathways were identified as the key metabolic pathways in this study. The matched metabolites in these two key pathways were L-pyroglutamic acid, pyroglutamic acid, spermidine, and acetylcholine, respectively, and phosphocholine.

### 3.5. Correlations Between Differential Bacterial Taxa, Fecal Metabolites, and Behaviors

**Figure 5:**
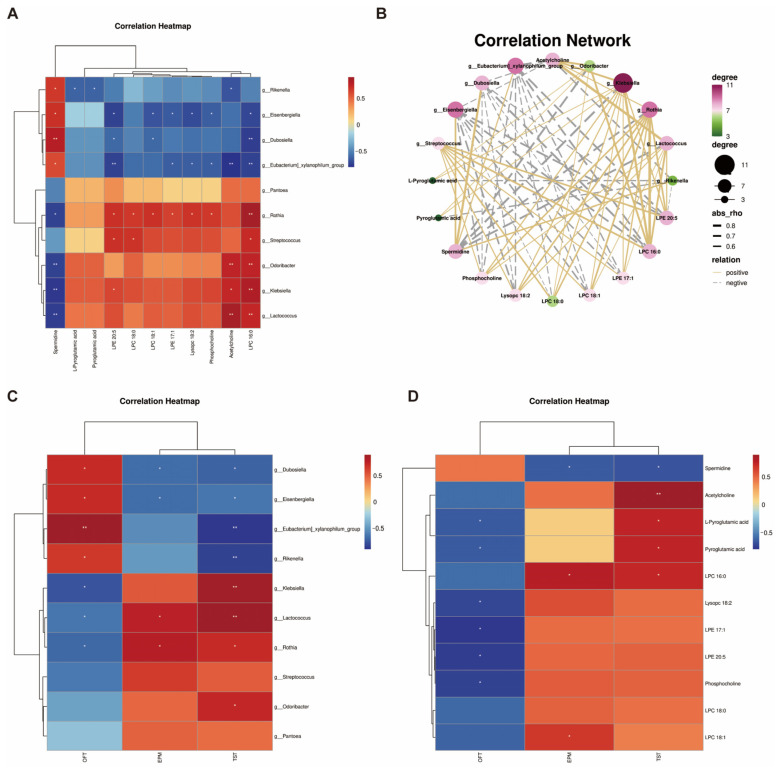
Correlation analysis between differential genera, metabolites, and behavioral indicators. (**A**,**B**) Correlation heatmap and correlation network between the top 10 genera with *p*-values less than 0.05 and metabolites in glycerophospholipid metabolism pathway and glutathione metabolism. (**C**) Correlation heatmap between the top 10 genera with *p*-values less than 0.05 and behavioral indicators. (**D**) Correlation heatmap between metabolites in glycerophospholipid metabolism pathway and glutathione metabolism and behavioral indicators. All data are presented as mean ± SEM. * *p* < 0.05, ** *p* < 0.01.

We performed a Spearman correlation analysis of the top 10 genera with *p*-values less than 0.05 with metabolites matched to two key metabolic pathways, glycerophospholipids metabolism and glutathione metabolism, and found that they were correlated to varying extents. *g_ Rothia* was positively correlated with seven glycerophospholipid metabolites and negatively correlated with spermidine, while *g_ Eubacterium]-xylanophilum-group* and *g_ Eisenbergiella* were negatively correlated with six glycerophospholipid metabolites and positively correlated with spermidine. In contrast, spermidine was positively correlated with *g_ Rikenella*, *g_ Eisenbergiella*, *g_ Eubacterium]-xylanophilum-group*, and *g_ Dubosiella*, and negatively correlated with *g_ Rothia*, *g_ Odoribacter*, *g_ Klebsiella*, and *g_ Lactococcus*. LPE20:5 was negatively correlated with *g_ Eisenbergiella*, *g_ Eubacterium]-xylanophilum-group*, and *g_ Dubosiella*, and positively correlated with *g_ Rothia*, *g_ Streptococcus*, and *g_ Klebsiella*. Acetylcholine was positively correlated with *g_ Rikenella*, *g_ Eubacterium]-xylanophilum-group*, *g_ Odoribacter*, *g_ Klebsiella*, and *g_ Lactococcus*. LPC16:0 was negatively correlated with *g_ Eisenbergiella*, *g_ Eubacterium]-xylanophilum-group*, *g_ Dubosiella,* while positively correlated with *g_ Rothia*, *g_ Streptococcus*, *g_ Odoribacter*, *g_ Klebsiella,* and *g_ Lactococcus*. *g_ Rikenella* was negatively correlated with pyroglutamic acid (Figure 5A,B).

Further analysis showed a correlation between some parts of the genus and metabolites with behavioral indices in mice. *g_ Dubosiella*, *g_ Eisenbergiella*, *g_ Eubacterium]-xylanophilum-group*, and *g_ Rikenella* were positively correlated with total distance in OFT and *g_ Klebsiella*, *g_ Lactococcus*, and *g_ Rothia* were negatively correlated with total distance. *g_ Dubosiella* and *g_ Eisenbergiella* were negatively correlated with anxiety indices in EPM, *g_ Lactococcus*, and *g_ Rothia* were positively correlated with anxiety indices. *g_ Dubosiella*, *g_ Eisenbergiella*, *g_ Eubacterium]-xylanophilum-group,* and *g_ Rikenella* were negatively correlated with immobility time in TST and *g_ Klebsiella*, *g_ Lactococcus*, *g_ Rothia*, and *g_ Odoribacter* were positively correlated with immobility time (Figure 5C).

L-pyroglutamic acid, pyroglutamic acid, lysopc18:2, LPE17:1, LPE20:5, and phosphocholine were negatively correlated with total distance. LPC18:1 and LPC16:0 were positively correlated with the anxiety index. Acetylcholine, L-pyroglutamic acid, pyroglutamic acid, and 1-palmitoyl-sn-glycero-3-phosphocholine were positively correlated with immobility time, whereas spermidine was negatively correlated with anxiety index and immobility time (Figure 5D).

## 4. Discussion

In the present study, we confirmed that CSD could induce anxiety/depressive-like behaviors in female mice. Moreover, CSD disrupted the colonic mucosal barrier, caused dysbiosis of gut microbiota, and altered its metabolites. Briefly, we found histological changes and a suppressed number of goblet cells in the colon of SD mice, reduced levels of beneficial bacteria and increased levels of harmful bacteria in the feces of SD mice, as well as altered KEGG metabolic pathways associated with inflammation and oxidative stress. In addition, Spearman correlation analysis indicated that some differential metabolites and genera may be significantly associated with anxiety/depression-like behavior in mice. Our results suggest that gut microbiota may be involved in SD-induced anxiety/depression-like behaviors by influencing fecal metabolites.

Current research has mostly focused on the effects caused by acute SD on male mice. However, diversely modern lifestyles have led to significant changes in sleep duration. For example, excessive use of electronic devices, excessive intake of caffeine, nicotine, or alcohol, night-shift work, pregnancy and lactation in women, and increased health problems and emotional stress all affect the sleep duration and quality of modern people [22,23]. All of the above factors are a long-term, chronic process and can create a vicious cycle and ultimately lead to anxiety disorders or depression, from which it is difficult to recover, so we aimed to investigate the effects of CSD on gut microbiota and mood, as well as on female mice.

Mammalian sleep consists mainly of rapid eye movement (REM) sleep and non-REM (NREM) sleep. NREM sleep helps to enter the sleep state (loss of somatic sensation and fading of consciousness), while REM sleep is mainly involved in mood regulation [16]. When using the MMPM, mice fall into the water and wake up when they transition from NREM to REM sleep due to the relaxation of muscles throughout the body. Rodents are naturally fearful of water, so they will keep themselves awake to avoid falling into water so that we can deprive their REM sleep [17]. Previous studies have shown that 6 h of SD was sufficient to induce anxiety-like behavior in mice, as evidenced by the OFT and the EPM [16]. In another study, 7 days of SD similarly decreased the total behavioral score of the OFT and increased the immobility time of the FST and the TST, which means 7 days of SD induced depression-like behavior in rats [24]. The decrease in total distance, central square duration, and entries into the center in OFT presented less locomotion and exploration. These indexes are often used to assess anxiety behavior together with EPM indexes. Although there are certain limitations, EPM is still the gold-standard method for assessing anxiety-like behavior in rodents and is widely used in sleep-deprived rodent models. Usually, mice with anxiety-like behavior will stay longer in the closed arms because they represent a safe environment, which indicates a decrease in locomotion. The reduced number of entries in the open arms indicates a decrease in exploratory behavior. However, anxiety disorder is a mental disease with complex symptoms; mania and impulsivity are also among the symptoms. In a meta-analysis of the effects of SD on anxiety-like behavior, we found that animals with impulsivity or mania spent more time in the open arms, presenting increased locomotor and exploratory behavior [25]. Thus, it is not a surprise that in our results of EPM, we found that the time spent in the open arms by SD mice was significantly longer than controls, which is consistent with the irritable phenotype we observed. However, we also found that the entries into the open arms were less than controls, which may indicate that SD mice stayed in the open arm for a long period but were not actually moving, which may indicate a decrease in locomotion and match with the OFT results. The anxiety index was calculated according to the formula of Suresh Konakanchi et al. SD mice had a higher anxiety index than controls [1]. There is a view that cognitive deficits are a phenotype of anxiety [26]. In our result, the recognition index of SD was decreased, which means CSD could influence their short-term cognition and memory. Finally, the FST and TST can reflect the desperate state of depression. In our results, the immobility time of TST was significantly increased, while in FST, the immobility time was increased without a statistically significant difference, but they could still indicate the depression-like behavior of SD to some degree.

Many studies have shown that there is bidirectional communication between the brain and the gut, and changes in gut function may occur even before CNS symptoms become apparent. Microbes in the mammalian gut communicate with the CNS via the enteric nervous system and can alter endocrine and immune signaling, and correspondingly, the CNS can influence the gut microbiota through corresponding pathways [27]. Intestinal epithelial cells (IECs) form a barrier to protect the intestine from harmful luminal ingredients. Dysbiosis of the gut microbiota may disrupt the epithelial integrity and trigger an inflammatory reaction due to the infiltration of potentially harmful components and metabolites [28]. Similarly, previous studies have proved that SD can change the composition of gut microbes and decrease the antioxidant ability, down-regulate the anti-inflammatory cytokines, and up-regulate pro-inflammatory cytokines in mice, which leads to colonic mucosal injury, including a reduced number of goblet cells, proliferating cell nuclear antigen-positive cells, expression of MUC2, and tight junction proteins [6,24,29]. In our study, we found alterations in the colonic mucosa of mice in the CSD group, as evidenced by epithelial cell hyperplasia, decrease in goblet cells, and glandular atrophy, which may lead to disruption of the intestinal barrier and increased intestinal permeability, making the intestine more susceptible to bacteria and their antigens.

In 16SrDNA sequencing, we found a significant increase in the abundance of *g_ Rothia* and a significant decrease in the abundance of *g_ Dubosiella* in SD mice, a change that can be seen in the chronic mild unpredictable stress (CUMS) rat model of depression [30,31]. In addition, the researchers found a significant increase in *g_ Odoribacter* in the cecum microbiota of stress-exposed mice, while it has also been shown that increased *g_ Odoribacter* was found in the feces of mice with acute colitis [32,33]. In our study, an increase in *g_ Odoribacter* was similarly found, as well as an increase in the relative abundance of the common pathogenic bacteria *g_ Streptococcus* and *g_ Klebsiella*. Coincidentally, *g_ Streptococcus*, *g_ Klebsiella*, and *g_ Odoribacter* was found to be higher in the feces of patients with major depression (MDD), which is strikingly consistent with our findings [34,35]. As CSD is also a way to cause stress, we speculate that CSD may share some of the mechanisms of gut microbiota disruption with CUMS-induced depression-like behavior. Additionally, Rothia, Odoribacter, Streptococcus, Klebsiella, and Pantoea are all pathogenic bacteria associated with humans [34,35,36,37]. The relative abundance of probiotic *g_ Lactococcus lacti* was increased in the feces of mice in the SD group compared to the control group. There is a study that found oral administration of *g_ Lactococcus lacti*, a subspecies of Lactococcus, attenuated DSS-induced colitis [38], and the percentage of Lactococcus in the feces of ulcerative colitis (UC) cancerous mice was increased after probiotic treatment [39]. We also noticed that oral administration of Lactococcus lacti improved symptoms in mice with depression induced by chronic stress and LPS [40,41]. Probiotics are now known to promote intestinal homeostasis not only by blocking pathogenic bacteria but also by increasing the integrity of the intestinal epithelial barrier, stimulating the innate immune response and balancing the production of inflammatory cytokines [42]. Thus, in our result, we hypothesize that there may be a phenomenon of increased protection due to such a chronic process. In addition, in our study, we found an increase in the relative abundance of *g_ Pantoea*, a rare opportunistic pathogen that is most commonly found in nosocomial infections, and susceptible populations include infants, young children, postoperative, and other immunocompromised populations [37]. The present study also found a decrease in the relative abundance of *g_ Eubacterium]-xylanophilum-group*, which was found in previous studies to be the bacterium that produces branched-chain amino acids (BCAAs) and short-chain fatty acids (SCFAs), which are known to contribute to intestinal health and can modulate intestinal immune responses and maintain intestinal barrier integrity [43,44]. The beneficial bacterium *g_ Rikenella* was reduced, which may indicate that the intestines of SD mice may be in an inflammatory state [45]. Additionally, the relative abundance of *g_ Eisenbergiella* was reduced. *g_ Eisenbergiella* is a bacterium that produces butyrate in the intestine; butyrate is one of the sources of energy for the colonic epithelium, and the decrease in the relative abundance of *g_ Eisenbergiella* in the feces of the SD group may affect the energy supply of colonic epithelial cells [46]. The above results suggest that the dysbiosis led to difficulties in maintaining the integrity of the colon barrier and impaired defensive functions, leaving the colon in a state of inflammation and susceptibility to pathogenic bacteria.

Gut microbiota can exert their influence on an organism through metabolites, so we used LC-MS untargeted metabolomics to detect fecal metabolites in mice. In our result, we identified the glycerophospholipid metabolic pathway and glutathione metabolic pathway as key pathways. First, glycerophospholipids are one of the major components of cell membranes, which help to recognize protein signals on cell membranes and can be mainly classified into phosphatidylcholine (PC), phosphatidylethanolamine (PE), phosphatidic acid (PA), and phosphatidylinositol (PI), which are the sources of substrates for a variety of lysophospholipids, such as lysophosphatidylcholine (LysoPC), lysophosphatidylethanolamine (LysoPE), lysophosphatidic acid (LysoPA), and lysophosphatidylinositols (LysoPIs). Among them, LysoPC is a biologically active pro-inflammatory lipid, and blood levels of lysoPC and/or lysoPE are elevated under many pathophysiological conditions (e.g., inflammation or oxidative stress) [47,48,49]. LysoPC disrupts the colonic barrier by increasing the inflammatory response and impairing tight junctions [50]. We identified a variety of glycerophospholipids, including Lysopc (18:2), LPC (18:0), LPC (18:1), LPE (17:1), LPC (16:0) and LPE (20:5). Glycerophospholipid levels were significantly increased in the feces of SD mice compared to controls, suggesting that the intestines of SD mice may be in an inflammatory state. Meanwhile, phosphcholine was significantly elevated in the glycerophospholipid metabolic pathway and could be converted to phosphatidylcholine (PC) through a series of enzymatic reactions, and PC could release LysoPC in the presence of phospholipase A2 (PLA2) [49]. Previous studies have shown that glycerophospholipid metabolism is disturbed in the colonic lipidome of CSDS depression model mice and correlates differentially and significantly with anxiety/depression-like behaviors [51]. Interestingly, glycerophospholipids are key components of neuronal membranes and myelin in the brain, as well as major regulators of synaptic function. In a study of a nonhuman primate model of depression, it was found that the gut microbiota and hippocampal glycerophospholipid metabolism were disturbed in depressed monkeys, which could lead to depression and anxiety disorders, and most of the polyunsaturated fatty acids used for brain neuronal membrane glycerophospholipid synthesis originated from the gastrointestinal tract, suggesting that the MGB axis plays an important role in it [52]. On the other hand, in glutathione metabolism, we enriched two metabolites, 5-oxoproline (5-OP) and spermidine. 5-OP, also known as pyroglutamic acid, is an important component of the glutathione cycle, and it has been demonstrated that 5-OP accumulates in glutathione synthetase deficiency and that subcutaneous injections of 5-OP into rats promote oxidative stress in vivo by facilitating oxidative stress by promoting the oxidation of lipids and proteins [53,54]. The existing studies show that 5-OP induces oxidative stress in rat brain tissue, rat cardiomyocytes, and cardiomyocytes of human embryonic origin [55]. In contrast to elevated fecal levels of 5-oxoproline in patients with irritable bowel syndrome (IBS), 5-OP was reduced in the feces of Parkinson’s patients, but that is sufficient to suggest that altered fecal levels of 5-OP are associated with intestinal health as well as brain disorders [56,57].

In our study, Spearmen correlation analysis showed that 5-OP was associated with depressive-like behavior, but whether increased fecal 5-OP levels can induce colonic oxidative stress in mice needs to be further investigated. Spermidine is a natural autophagy inducer and anti-aging compound with cardioprotective and neuroprotective effects and also inhibits the expression of pro-inflammatory cytokines. Spermidine can be obtained orally not only from exogenous dietary sources but is also produced by colonic microbiota and macrophages. It has been proven that spermidine can support colonic epithelial proliferation and increase intestinal tight junctions and the mucosal barrier to play a protective role in intestinal barrier function [58,59,60]. In our study, the increase in glycerophospholipids and 5-OP and the decrease in spermine may have led to a state of oxidative stress and inflammation in the colons of SD mice. In addition, we noted that in the correlation analysis between enterobacteria and metabolites, most of the beneficial bacteria were negatively correlated with glycerophospholipids and positively correlated with spermidine, while the opposite was found for the harmful bacteria. As for the correlation analysis between enterobacteria and behavior, most of the beneficial bacteria were positively correlated with total distance in the OFT and negatively correlated with immobility time in the FST and hanging TST, while the opposite was found for the harmful bacteria. Finally, in the correlation of metabolites of the glutathione metabolic pathway and glycerophospholipid metabolic pathway with behavioral tests, glycerophospholipids and 5-OP were negatively correlated with the total distance of the OFT and positively correlated with immobility time. The results of the analysis of the three correlations were consistent with each other.

In conclusion, by comparing the species diversity and relative abundance of gut microbiota in the two groups, we found that although SD did not alter the diversity or abundance of the gut microbiota in mice, the structure of the microbial flora changed significantly; for example, the beneficial bacteria *g_ Rikenella*, *g_ Eubacterium]-xylanophilum-group*, *g_ Eisenbergiella* decreased in relative abundance and harmful or conditionally pathogenic bacteria *g_ Rothia*, *g_ Odoribacter*, *g_ Streptococcus*, *g_ Klebsiella*, and *g_ pantoea* increased in relative abundance, suggesting that the above microbial flora may play a key role in intestinal damage caused by SD. The changes in the metabolites of the microbial flora were in the direction of inflammation and oxidative stress, which could lead to the disruption of the intestinal barrier and had a correlation with anxiety and depression-like behaviors. However, the mechanisms involved need to be further investigated.

There are several limitations of this study. [1] Our study only focused on changes in fecal metabolomics in the colon and did not consider the brain regions associated with anxiety disorders and depression; additional studies on brain regions associated with emotion regulation (e.g., the hippocampus and prefrontal cortex) are needed in the future [2]. In addition, the enteric nervous system can contact the central nervous system via the vagus nerve, and signals from the gut can pass through the enteric nervous system (ENS) to the vagus nerve, which afferents signals to the nucleus of the solitary tract (NTS) and the dorsal nucleus of the middle suture (DRN). These regions can then interact with regulatory emotional brain networks [27,61]. Therefore, to elucidate the mode of action of the MGB axis, future studies should focus on the vagus nerve to determine whether gut microbial metabolites influence brain emotion regulation through the vagus nerve [3]. Our study used 16S rDNA sequencing, which has limited resolution, and higher resolution methods should be used to further investigate the species and functions of bacteria associated with anxiety and depression; the roles of differential flora and fecal metabolites need to be verified by further experiments.

## 5. Conclusions

In conclusion, our study showed that CSD induced anxiety/depressive-like behaviors in mice, leading to alterations in gut microbiota and fecal metabolites, as well as disruptions in gut barrier integrity. Moreover, there were significant correlations between some of the differential genera, metabolites, and behaviors, which may indicate that the gut microbiota could be an important mediator of intestinal homeostasis. Our results showed that CSD caused a decrease in the relative abundance of intestinal probiotics. As for the future directions, we plan to collect stools from people who are experiencing CSD and analyze the microbiota composition to compare with our findings. Additionally, our results showed that CSD caused a decrease in the relative abundance of probiotics. Therefore, we aim to investigate whether probiotic supplementation could restore the intestinal damage caused by CSD or ameliorate anxiety/depression-like behaviors in future experiments.

## Figures and Tables

**Table 1:** Results and trends in the identification of glycerophospholipids.

No.	Ion Mode	Name	RT [min]	Lipidmaps_ID	*m*/*z*	Trend	VIP	*p* Value	AUC
1	−	LPC (16:0)	9.406	LMGP01050113	540.33057	↑	1.93	0.0021	0.97
2	+	LPC (18:2)	9.545	LMGP01050137	520.34058	↑	1.67	0.0131	0.87
3	−	LPC (18:0)	10.298	LMGP01050076	568.36273	↑	1.58	0.0235	0.83
4	−	LPC (18:1)	9.391	LMGP01050138	566.34705	↑	1.55	0.0266	0.87
5	+	LPE (17:1)	8.87	LMGP02050008	466.29416	↑	1.49	0.0424	0.87
6	+	LPE (20:5)	8.2	LMGP02050053	500.27875	↑	1.41	0.0432	0.87

Note: +: Positive ion mode, −: Negative ion mode, Lipidmaps: Lipid database.

## Data Availability

Data supporting the findings of this study are available upon request from the corresponding authors.
